# An econometric analysis for the determinants of flight speed in the air transport of passengers

**DOI:** 10.1038/s41598-023-30703-y

**Published:** 2023-03-20

**Authors:** Eric Eduardo de Almeida, Alessandro V. M. Oliveira

**Affiliations:** Aeronautics Institute of Technology, Center for Airline Economics, São José dos Campos, 12228-900 Brazil

**Keywords:** Aerospace engineering, Civil engineering

## Abstract

Accurately determining an aircraft's flight speed is crucial for optimizing airline performance, as it directly impacts factors such as fuel consumption and emissions. Flying at speeds higher than what is recommended by the manufacturer can result in increased fuel burn. However, flying at slower speeds may lead to longer flight times and competitive disadvantages for airlines as passengers typically prefer shorter travel times. This study empirically investigates the driving forces in the decision-making process of airlines when setting flight speeds to reduce costs while maintaining the quality of service provided to customers. We develop econometric models of planned flight cruise speed and actual mean flight speed. We analyze a vast amount of data, comprising millions of domestic flights within Brazil. Our results allow for policy recommendations that identify opportunities for improvements in airline flight operations optimization, with implications for the environmental footprint of commercial aviation.

## Introduction

Airline flight operations are a complex decision-making process subject to several factors. Weather, maintenance, utilization, airport rules, crew and passenger connections, and pre-flight tasks like fueling, cargo handling, and provisioning are among the many factors that can affect flight conditions. These activities may generate delays in the dispatch of the aircraft. In addition, at airports with high passenger and aircraft traffic and operating close to their capacity, delayed arrivals or departures may incur penalties for airlines^1^. Airlines must consider all these factors in their strategic and tactical flight planning, with possible impacts on the choice of cruise speed for a given flight.

The planned and the actually performed speed has a direct impact on the airline expenses and operations associated with the flight. One can mention, for example, fuel consumption, which increases as aircraft fly at higher speeds, closer to the speed of sound, or is attenuated, as aircraft flies at a lower speed, close to its economic speed. Since fuel costs represent one of the main costs for airlines^2^, an initiative to reduce them becomes an airline's interest. This is intensified in the scenario where airlines seek to reduce carbon emissions. Another direct impact is on the flight’s duration, which increases as aircraft flies at lower speeds. In this case, there are time-related costs that gain importance as the flight time increases.

Besides fuel and time-related costs, aircraft speed can indirectly impact passenger satisfaction as it is related to flight duration. According to Kang and Hansen^3^, passenger may prefer shorter flights instead of longer ones on the same route. Additionally, another aspect of satisfaction is flight punctuality, measured through OTP (On Time Performance). This factor has the potential to affect the image of airlines, as passengers may prefer companies that have a better history of punctuality to those that have a history of delays^4^. In this sense, the aircraft speed is indirectly related to factors of market competition. Thus, the level of competition between airlines may influence the determination of aircraft cruise speeds.

Considering that flight speed is inherent to airlines operating costs, the low-cost business model emerges as a factor to be examined more closely. Is there any way to identify a company's business model by observing its practices regarding flight speeds? Given that aircraft speed influences fuel consumption, and at the same time, low-cost carriers (LCCs) seek to reduce their costs, one can assume that a LCC flies at more economical speeds. However, it is also a LCC characteristic to increase their aircraft utilization^5–7^, so that higher cruise speeds would favor this aspect. Thus, the knowledge of flight practices could be an additional tool to understand airlines' business models.

In the context where cruise speed plays a role in airlines' costs, market competition, and business models, this study proposes an econometric model that: (1) analyzes whether airlines plan and perform more economical flight speeds as a tool for savings in scenarios of higher fuel costs; (2) tests if competitiveness factors influence the determination of cruise speed, as it can influence the punctuality policy of airlines; (3) tests if there is a differentiated behavior of an LCC company regarding the determination of aircraft speed. Does this company use slower speeds to achieve fuel savings, or does it prefer policies of increasing aircraft utilization?

To study the aircraft speed, we observed it from two different perspectives. The first one is the cruise speed performed by the aircraft. The actual speed is subject to several random unforeseen situations such as delays, need for route deviation, emergencies, weather conditions, etc. Thus, one cannot observe this aspect but may estimate it through the mean speed. The second approach is the cruise speed planned by airlines for flights, as informed in the Repetitive Flight Plan (RPL). Employing the planned cruise speed in the analysis can contribute to the literature since it reveals the ideal scenario pre-defined by airlines regarding the cruise speed.

This paper is composed by the following sections. Section “Literature review” describes literature review about the aircraft flight speed, and the themes related to it, such as fuel consumption, and market competitiveness as low-cost carriers’ practices. Section “Research design” presents the methodology applied in for this analysis. Section “Results and discussion” presents the results and discussions. Section “Conclusion” presents the conclusions.

## Literature review

### The flight speed determination problem

One of the main study fronts in the literature about flight speed is on the definition of speeds for recovery of consecutive flights, i.e., the effort to avoid propagation of delays when they occur in previous flights. The scenario studied by Aktürk et al.^8^ considers that flight recovery commonly involves increasing cruise speed to reduce delays. However, this strategy implies higher fuel consumption, resulting in environmental impacts due to higher emissions. In this scenario, Aktürk et al.^8^ propose a model that includes cruise speed as a factor to be optimized in the delay recovery process, along with the costs resulting from the delay. Skaltsas^9^ describes this practice of increasing flight speed to recover consecutive flights as a factor that explains the relationship empirically found between higher punctuality and flight distance.

Delays occurring on the ground also can influence speed control, for example, when there is congestion at the destination airport. In such situations, aircraft must remain in flight until air traffic control authorizes it to land. Thus, instead of flying over the landing airport, Delgado and Pratz^10^ evaluate a model that reduces the cruise speed so that the operation absorbs the delay and reduces fuel consumption. In a similar context, where the constraint is a fixed time of arrival at the destination, Franco and Rivas^11^ develop a model to optimize flight speed, considering fuel consumption and time-related costs.

Aircraft speed also impacts and is impacted by air traffic control. Cafieri and Durand^12^ and Cafieri and d’Ambrosio^13^ develop models of aircraft deconfliction, that is, the problem of detecting and solving the situation in which airplanes share the same airspace and are potentially in trajectory conflict by being insufficiently separated from each other, horizontal or vertically. In events like this, air traffic control can determine the alteration of airplanes’ heading or altitude, or, as proposed by the authors, change the airplanes' speed as little as possible to remove them from the conflict situation.

Relating flight speed and flight schedule, Şafak et al.^14^ propose a model that uses, among other factors, higher cruise speeds to free up time when there is a need to insert unforeseen flights into a pre-defined schedule. In this model, an optimal cruise speed maximizes airline profitability, compensating for financial and environmental costs due to higher speeds.

The mentioned studies develop models for defining the optimal cruise speed in abnormal situations to airline operations, such as delays in departure or arrival, to accommodate changes in flight schedules, or to resolve conflicts in the airspace. All these papers highlight the importance of flight speed for airline operating costs. However, they do not describe the airlines' speed policies under normal conditions. We observe a lack of studies in the literature involving econometric modeling to evaluate the factors that influence the choice of flight speed, considering the planned cruise speed, or its estimative such as the mean speed. The following topics presents how the literature treats factors related to flight speed.

### Fuel price and flight speed

Fuel cost is one of the most relevant components of airlines’ operating costs^2^. According to Edwards et al.^15^, fuel costs accounted for 32% of airlines' global operating expenses in 2014, five times higher than in 2003. For Şafak et al.^14^, the importance of this component increases as its price increases, and there is a perception in the airline industry that cruise speed selection has a significant impact on airline profits.

In addition to fuel purchase itself, there are other costs associated with fuel consumption, such as the carbon offsets defined by ICAO's CORSIA (Carbon Offsetting and Reduction Scheme for International Aviation), which requires airlines to buy carbon credits to compensate for their emissions. Thus, as emissions are related to fuel consumption, the carbon offsetting program increases fuel-related costs for airlines. This scenario makes companies adopt measures to reduce consumption, such as replacing old aircraft with modern ones, and implementing strategies to reduce the consumption of existing aircraft, such as taxiing with one engine, loading less reserve fuel to reduce weight, and adopting lower cruising speeds^16^.

Variations in fuel price impact companies in several ways, including their network structuring and aircraft operations. McConnachie et al.^17^, by analyzing the impact of fuel prices on airline operations, observed through data analysis and interviews that there were significant changes in operational aspects, such as a reduction in mean flight speeds, concomitantly with an increase in fuel price in the United States between 2004 and 2011, which may have led to an improvement in aircraft energy efficiency.

Typically, the behavior of fuel consumption against aircraft speed is a parabolic curve in which there is a speed where the fuel cost is minimum^18^. This speed is called long-range speed. At values below it, as in the climb and approach phases, the fuel consumption is higher. On the other hand, in cruising flight with aircraft speed above the long-range speed, the faster the aircraft, the higher the fuel consumption and, therefore, the fuel-related cost.

In addition to fuel-related costs, which increase as aircraft fly at higher speeds (above long-range speed), there are time-related costs such as those with the crew, aircraft maintenance, etc. The sum of fuel-related costs, time-related costs, and fixed costs makes the total flight cost. To determine the speed at which the total cost is minimum, the cost index (CI) is a tool available on most commercial aircraft since the 1970s^15^ and which has the potential to contribute to the measures to reduce fuel consumption. The CI represents the cost per unit of time CT divided by the cost per unit of fuel CF for a specific flight. By using the CI, if time-related costs are high, the flight is expected to be faster to reduce them, even if it means higher fuel consumption. On the other hand, if fuel cost is the main driver of operating costs, then the cruising speed will be lowered to minimize fuel consumption^19^.

In the literature, several studies utilize the CI to achieve more efficient operations. Edwards et al.^15^ study how the CI may reduce CO_2_ emissions. According to the authors, by balancing time with fuel costs, the CI controls aircraft speed to achieve the most economical flight time. The impact on the amount of CO_2_ emitted occurs since higher speeds result in higher fuel consumption. In the same direction, Tian et al.^20^ propose a model in which environmental costs are considered additionally to fuel and time-related costs. In this model, the cruising speed is one of the variables to be optimized to minimize the total operating cost.

Deo et al.^19^ use the cost index as a variable to assess aircraft refueling strategy. This study analyzes the possibility of aircraft making stops to refuel at intermediate airports so that the airplane flies with less fuel, reducing its average weight. However, as this process can increase flight time, the cost index is used to prevent time-related costs from overlapping the savings achieved with the refueling strategy.

The studies discussed in this section focus on developing methodologies that allow airlines to optimize flight speed, respecting some constraints. However, there is no measure of how airlines change aircraft speeds in actual operations when there are fuel price variations. In this context, this research examines the behavior of airlines regarding flight speed when there are changes in fuel prices, i.e., it tests the hypothesis that airlines adjust their operation about flight speed planning, using the cost index, as an alternative to reduce operating costs.

### Competition and flight speed

Flight speed determines the duration of the flight, which composes the block time. The block time is the elapsed time between the instant the aircraft leaves the departure gate at the origin airport and the instant the aircraft parks at the arrival gate at the destination airport^21^.

Several studies approach the composition of block time. Through an econometric analysis, Coy^21^ evaluates the factors that influence block time. According to the author, some variables like the population of the origin and destination cities, arrival time, airport use, weather conditions, and their interactions with air traffic have a significant influence on block time formation.

The strategy for block time formation may be a result of market factors. The literature observes that the practice of airlines to include an additional time in the block time to improve their performance concerning the OTP (On-Time Performance) and thus improve their image with consumers. Forbes et al.^22^ note that airlines can improve their OTP by increasing the time between takeoff and scheduled landing. Eufrásio et al.^23^ decomposes the extra time added to the scheduled flight time between operational and strategic factors in the Brazilian air market and find evidence that reaffirms the existence of this practice. For Skaltsas^9^, a reliable schedule that reduces the propagation of delays through an airline's network increases customer satisfaction. However, despite the positive impacts of punctuality, which has the potential to attract passengers who reward lower chances of delay, as noted by Prince and Simon^4^, adding extra time to flight planning can mean an increase in the total flight time of a trip.

For Kang and Hansen^3^, from the passengers' point of view, shorter flights may be preferable to longer flights on the same route and overestimating the SBT (Scheduled Block Time) may result in a loss of competitiveness for an airline. In the same direction, Skaltsas^9^ suggests that shorter flights may become a market advantage, especially on routes of intense competition. This is because, when passengers buy tickets, the system may list the flights according to their scheduled duration, showing preferably on screen the shortest ones. Several studies seek to relate the block time to market competition. The focus is to verify the behavior of companies concerned with their image associated with the frequency of delays. Kang and Hansen^3^ find evidence that, on highly competitive routes, airlines tend to increase the block times. Conversely, Prince and Simon^4^ and Fan^24^ suggest that airlines reduce scheduled travel time on less competitive routes, making them more vulnerable to delays. All these studies observe the block time. They do not analyze if and how aircraft speed contributes to airlines' OTP indexes.

Based on interviews with airlines in the USA, Kang and Hansen^3^ observe that early arrivals increase customer satisfaction by providing extra time for connecting passengers. This is a way to exceed passengers' expectations, especially when the crew announces it to passengers in the final phase of the flight. Deshpande and Arikan^25^ point out, however, that the practice of early arrival can induce costs.

It is notable in the literature that defining the flight block time and its impacts on airlines' image is widely studied. However, such studies do not inspect the role of flight speed in scheduling. Similarly to the block time formation strategy, we question if airlines choose flying faster on routes with more competition to improve their OTP, and fly at lower speeds on less competitive routes to reduce fuel consumption. Another hypothesis we test is whether airlines plan higher cruise speeds to avoid possible delays on routes with higher competition, that is, where there is a greater possibility of delays to impact the company's image to passengers.

### Flight speed and low-cost carriers

Low-cost carriers are those that stand out for adopting a market strategy that aims to retain their customers by offering low fares but reduced additional services^7^, i.e., the passenger pays only for the basic product, which is the transport itself^26^. To be able to offer the lowest fares, LCCs prioritize practices to reduce operating costs and greater exploitation of their assets, such as: reduction of space between seats, single class configuration, reduced in-flight service or with extra charges, point-to-point network (when flights are carried out directly between the cities of origin and destination, without connection), operation in secondary airports, whose fees are lower, fleet standardization, etc.^7,26^.

In contrast to LCCs, another commonly studied business model is the full-service carrier (FSC). This model offers to passengers a higher level of service, including in-flight entertainment, meals, more space between seats, and business classes possibility, among others. Regarding flights operation, according to Gillen^27^, FSCs generally utilize the hub-spoke system, in which airlines distribute flights to the cities of demand from a hub airport. Consequently, these companies are predominant in the hub airports, where the movement of passengers and aircraft is high.

High aircraft utilization is one of the key factors for success in the LCC operating model. The standard measure of aircraft productivity is its daily utilization, i.e., the average daily hours each aircraft operates in the fleet. According to Alamdari and Fagan^5^, LCCs achieve high utilization rates by performing lower TAT (Turnaround Time) than traditional airlines (FSCs), thus making it possible to increase the number of segments flown daily. Morrell^6^ and Gillen^7^ state the same, adding that the TAT is lower due to a reduced catering service. Also, operating in less congested secondary airports favors a more efficient use of ground staff and aircraft. Morrell^6^ also highlights that LCCs encourage high productivity of their pilots by reducing the fixed part of their salaries and increasing the portion related to hours flown.

In this sense, one can expect that a company operating similarly to LCCs would consider flying faster to reduce the block time and to include more flights in a day of operation. On the other hand, as Singh et al.^2^ argue, low-cost carriers are pioneers in implementing policies to reduce fuel consumption and its expenses, consequently increasing profits. In the same direction, the results of Brueckner and Abreu^16^ demonstrate that, in the North American air market, aircraft operated by LCC companies are more efficient in terms of fuel consumption than others. Therefore, flying faster to increase aircraft utilization would imply an increase in costs, which contradicts the characteristics of low-cost companies.

The literature analyzes common characteristics of low cost-companies and how they have changed over time. However, there is a gap in the literature about the practices of LCCs related to flight speed. We intended to test if the predominant practice of low-cost carriers is the search for cost reduction through fuel savings or the high utilization of the aircraft.

## Research design

### Conceptual model

This study investigates some of the primary factors influencing the determination of flight speeds by airlines operating in the Brazilian domestic air transport industry. To achieve this objective, we utilize an econometric approach. This approach enables us to examine the presence of statistical associations between the variables being studied, as well as to isolate the impact of one variable while holding all others constant.

In our framework, the explained variable is the flight speed set by commercial airlines, analyzed from two perspectives: the planned flight cruise speed and the actual mean flight speed performed by airlines–hereafter SPDCRU and SPDMEA, respectively.

We identify key drivers of flight speed determination based on a literature review. We classify these factors into five categories: flight operations, flight delay management, airport operations, aircraft characteristics, and market and industry conditions. Some relevant aspects of flight operations that may influence the decision-making concerning flight speed determination are not directly observable by the researcher. These factors constitute the unobserved portion of the flight speed determinants. Below is a discussion of each flight time determinant category.

- Flight operations: involves overseeing and ensuring the safe and efficient functioning of an aircraft in all flight stages. These tasks encompass a variety  of responsibilities, such as arranging flight schedules, assigning aircraft and flight crews, ensuring aircraft maintenance and repair, and complying with all necessary safety and regulatory standards. The airlines commonly consider costs and competition conditions in this setting. For example, Mcconnachie et al.^17^, observed a reduction in mean speeds concomitantly with increases in fuel prices.
- Flight delay management: refers to identifying, assessing, and mitigating delays in flight operations. A delayed flight can cause a cascading effect on the entire schedule of a carrier and disrupt the plans of many passengers and airports, besides other airlines. Flight delay management is therefore a critical function of flight operations. One of the strategies of flight delay management is flight schedule recovery, which can include adjusting the flight schedules and speeds, besides rerouting flights to minimize the impact of delays. Prince and Simon^4^, Kang and Hansen^3^, Bendinelli et al.^28^, Eufrásio et al.^23^, and Calzada and Fageda^29^, among many others, investigate the association between market competition and airlines’ concern with punctuality.
- Airport operations: refers to the management and coordination of all activities performed at the endpoint airports of a flight. It involves many institutions besides the airline, including, among others, the ground handling of aircraft, passenger and baggage processing, air traffic control, and maintenance of the airport facilities and equipment. The primary goal of airport operations is to ensure that flights are conducted safely and efficiently, while also providing a high level of service to passengers and other airport users. On the other side, airport congestion and slot restriction policies can affect the efficiency of airport operations^30,31^.
- Aircraft factors: involves airplane-specific factors, such as weight, engine, aerodynamics, and generation, which are aeronautical engineering-related determinants of flight speed^18^. Oliveira et al.^32^ and Brueckner and Abreu^33^ assess the effect of payload and age of aircraft on their energy efficiency.
- Market and industry environment: refers to the state of competition between airlines, and factors that drive the expectations and dynamics of the air transport industry. It may involve consumer and technological trends, as well as pressures for carbon footprint mitigation, and broad aspects of cost competitiveness and business models of carriers (Singh et al.^2^, Brueckner and Abreu^16^, Oliveira et al.^34^, among many others).
- Unobserved flight speed determinants: in our econometric modeling, we aim at finding good and intuitive regressors associated with each of the categories raised above. However, in all of them, there are aspects not observed by the researcher in our study but are part of pilots' and airport agents' information sets. For example, flight trajectory particularities, weather and wind direction changes, air traffic control new procedures, among others. We will discuss below how we can incorporate some of these factors into the model through fixed effects interpreted as nuisance parameters.

**Figure 1:**
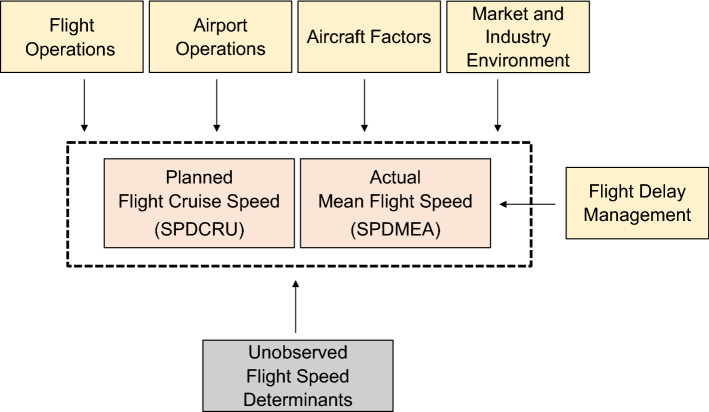
Conceptual model of flight speed determination.

Figure 1 synthetically presents our conceptual model.

### Data

Our data consist of detailed operational information of more than 6 million domestic passenger flights in Brazil scheduled between January 2009 and November 2022. The data set comprises 14 years, 5017 operating days, 1255 airport pairs, and a total of 813 million revenue passengers. We consider data from the three major carriers in the country, Latam (36% market share from Dec 2012 to Nov 2022; source: ANAC), Gol (33.7%), and Azul (29.7%). There are 726 different airplanes and 22 aircraft models–ICAO codes A20N, A21N, A319, A320, A321, A332, A339, A359, AT45, AT72, AT75, AT76, B38M, B733, B737, B738, B763, B777, E170, E190, E195, and E295).

The main data source is the country's airline regulator, National Civil Aviation Agency (ANAC), through the following online databases: 1. Active Scheduled Flight Historical Data Series (VRA/ANAC), 2. Air Transport Statistical Database, Microdata (ATSD-M/ANAC), and 3. Brazilian Aeronautical Registry (RAB/ANAC). To estimate the age of airplanes employed in each flight, we confronted RAB/ANAC's data with the information provided from the web sites planelogger.com, airfleets.net, jetphotos.com, and aviacaopaulista.com. These sources contain information by aircraft registration, like the date of the first flight. Concerning the fuel price, we collected data from the ANP (National Agency for Petroleum, Natural Gas and Biofuels) website.

For the regressands, we considered measures of *SPDCRU* and *SPDMEA*.

*SPDCRU* is the flight cruise speed planned by airlines, i.e., the cruise speeds declared in the repetitive flight plans (RPL). A repetitive flight plan (RPL) is a type of flight operations plan describing a series of frequently recurring, regularly operated individual flights with identical characteristics. Airlines are responsible for its development and submission to the Air Navigation Services (ANS)/Air Traffic Services (ATS) teams for retention and continued utilization. The main advantage of using this type of flight plan is that it can improve the efficiency of operations and lower costs by making the planning and management of resources by the airline more predictable. Furthermore, it enhances flight safety by allowing pilots and air traffic controllers to become familiar with the specific routes and procedures. The RPL contains information like days of validity, aircraft identification, aircraft model, origin and destination airports, departure and arrival times, flight declared cruise speed, altitude, and route. We collected RPL data for the period from February 2017 to July 2021.

*SPDMEA* is the mean actual flight speed performed by carriers. We estimate SPDMEA by using records of departure and arrival times of actual flights from the VRA/ANAC database and computing the Viencenty distance (*VDIST*) between the origin and destination airports. The Vincenty distance is a technique to calculate the geodesic distance between two locations on the Earth's surface, taking into account the Earth's irregular shape represented by an ellipsoid. It is particularly useful for small to medium distances, as it is a more precise method than the traditional Haversine formula. We compute *VDIST* by using the vincenty routine available for Stata^35^. With this method, it is possible to determine the distance between any two points on the Earth. The mean actual flight speed can be calculated by Eq. (1), where *FLTIME* is the difference between arrival and departure for each flight available in the database.

1 $$SPDMEA=\frac{VDIST}{FLTIME}.$$

By estimating the mean speed according to Eq. (1), we should be aware that the time between arrival and departure may include delays in taxiing or in the approach phase, for example, when the aircraft remains flying over the airport until it receives authorization to land. The effect of these situations is to reduce the calculated mean speed. Also, the traveled distance may be greater than *VDIST*, since the flight trajectory comprises segments that do not necessarily make a straight line. Besides, flights comprise a climb phase, where the aircraft accelerates, and an approach phase, where it decelerates, making the mean speed value to decrease as the routes are shorter. Given these limitations of the *SPDMEA* metric, we consider the mean actual flight speed computed from Eq. (1) as an alternative way to analyze the costs, operations, and market incentives of airlines to set their flight speed. We therefore combine our *SPDMEA* and *SPDCRU* analyzes to enhance our understanding of the research subject and to check for possible consistencies and inconsistencies between them. We also discard mean actual flight speeds outliers below the percentile 2.5, and above the percentile 97.5, so that atypical flights, i.e., those with duration much longer than the standard, do not affect the analysis' results.

To build our database, we merged different sources of information, namely RPL, ATSD-M/ANAC, and VRA/ANAC. The date and flight identification are the indicators utilized to join the information from planned and actual flights. Following this procedure, out of the more than 6 million flights extracted from the VRA (and ATSD-M/ANAC), we found 887 thousand correspondent observations of planned flight cruise speed from the RPL reports.

### Econometric model

Equation (2) contains our baseline econometric model.

2 $$SPD = {\beta }_{1}VDIST+ {\beta }_{2}VDIST2+ {\beta }_{3}MASS+ {\beta }_{4}FUELP+ {\beta }_{5}DELDEP+ {\beta }_{6}DELARR+ {\beta }_{7}CONG+ {\beta }_{8}MAXFREQ+ {\beta }_{9}SLOT+ {\beta }_{10}HUB+ {\beta }_{11}AGE+ {\beta }_{12}NEWMOD+ {\beta }_{13}GOL+ {\beta }_{14}AZUL+ {\beta }_{15}NCOMP+ {\beta }_{16}TREND+ {\beta }_{17}TREND\times PAND+ \omega ,$$

where *SPD* = {*SPDMEA*, *SPDCRU*}. The variables are described as follows:

#### Flight operations

*VDIST* is the vincenty distance of a given flight in kilometers (in logarithm); *VDIST2* is equal to *VDIST* squared. Expected signs: positive for *VDIST* and negative for *VDIST2*, to allow for increasing speed with flight distance, at a decreasing rate.

*MASS* is a proxy for the overall load factor of a flight. It is equal to the sum of passenger (multiplied by 75 kg), and baggage, cargo, and mail kilos (in logarithm, plus 1). This variable controls the fact that aircraft with higher load factor (greater weight) need to fly at higher speeds so that the lift force generated balances the weight. Expected sign: positive.

*FUELP* is equal to the mean fuel price over the 30 days previous to the flight (in logarithm). It is a pre-tax, deflated value. This variable allows to identify whether variations in fuel price exerts influence over the determination of flight speed. Airlines may adjust their aircraft cost-index when fuel price increases, requesting their pilots to perform more economical flights, possibly through lower flight speeds^17^. Expected sign: negative.

#### Flight delay management

*DELDEP* is the takeoff delay in minutes (divided by 10). With this variable, we control the behavior of airlines when there is a delay at the flight's origin. One can expect that mean speeds will be higher to recover the scheduled time in this situation, as predicted by Aktürk et al.^8^ and suggested by Ryerson et al.^31^. Expected sign: positive.

*DELARR* measures arrival delay in minutes (divided by 10). Ceteris paribus to *DELDEP*, an arrival delay means the flight had additional flight operations issues in its trajectory, due to weather conditions, winds, turbulence, or air traffic control and airport operations management. Expected sign: negative.

The hypotheses regarding *DELDEP* and *DELARR* dialogue with Brueckner and Abreu^33^, who test if fuel consumption increases in situations of delays. However, the authors attribute the higher consumption to a longer taxiing time or overflight waiting for authorization to land. There is no mention of the possibility of aircraft consuming more by flying faster to recover from delays.

#### Airport operations

*CONG* is a proxy to represent the level of congestion at an airport. It is measured by the maximum percentage of airport delays in one hour between the origin and the destination airport (in logarithm, plus 1). This variable differs from *DELDEP* and *DELARR*, which are flight-to-flight delays since it accounts for the level of airport delays when a specific flight is observed. Thus, the more delays at a given time at a specific airport, the greater the congestion. In a similar way as Eufrásio et al.^23^, who estimate the effect of airport congestion on block time formation, here we inspect the effect of congestion on planned cruise and mean actual flight speeds. Greater congestion at the destination airport is expected to induce lower speeds. Expected sign: negative.

*MAXFREQ* indicates the maximum number of flights, at a given time, between the airports of origin and destination (in logarithm). This variable represents the scale of operations at an airport. At airports with higher frequencies, where congestion is more likely, there is pressure for airlines to comply with flight schedules, relying on less time on the ground^23^. Thus, a possible way for carriers to keep their level of on-time performance is to employ higher flight speeds on these routes. On the other hand, a higher level of complexity of operations at the airport and its terminal area may impair flight times and decrease speed. Expected sign: undetermined.

*SLOT* is a dummy variable indicating whether there is a slot constraint in any of the origin and destination airports. The current slot-restricted, IATA level 3 airports in Brazil are São Paulo/SBSP and SBGR, Rio de Janeiro/SBRJ, Belo Horizonte/SBBH, and Recife/SBRF. However, other airports were slot-restricted in the past, due to transitory operational conditions and to the advent of tourism mega-events in the country. A slot-restriction policy is commonly employed by authorities to mitigate flight delays at high-traffic airports by imposing penalties on airlines that do not meet established requirements of on-time performance and the use of slots. This variable may have a different sign depending on whether speed is planned or accomplished. One can assume that airlines plan slower flights on routes containing these airports in a strategy like padding the block time. Regarding mean speed, it can be higher on these routes to avoid or cover delays^32^. Expected sign: undetermined.

*HUB* is a variable to observe the behavior of airlines in terms of speed at their hub airports. It is a dummy assigned with value 1 if any of the endpoint airports is connected with more than 20 destinations in a given airline network. With this algorithm, we are able to account for all airports in the country that have been officially declared as "hubs" by the studied airlines—for example, São Paulo/SBSP and SBGR for Latam and Gol, and Campinas/SBKP and Belo Horizonte/SBCF for Azul, among others. As with *SLOT*, operations are more complex at hub airports, and there may be congestion with a greater risk of delay^31^. Thus, it allows testing if planned cruise speed is lower on flights involving hub airports to form a buffer time and to cover delays. The mean speed may be lower due to longer waiting time for takeoff permission. Expected sign: Negative.

#### Aircraft factors

*AGE* is a variable to account for the effect of aircraft aging on flight speed. It is equal to the number of months since the date of the first flight of the aircraft to the date of the considered flight (in logarithm, plus 1). As an aircraft ages, the accumulation of structural repairs and modifications may lead to an increase in the aircraft's weight. In this scenario, older aircraft may tend to fly at higher speeds. Expected sign: positive.

*NEWMOD* is a dummy variable that accounts for the use of new-generation aircraft models by the airlines. We consider new-generation families the A320-Neo (ICAO codes A20N and A21N), operated by Latam and Azul, the 737-Max (B38M), operated by Gol, and the E195-E2 (E295), operated by Azul. These models include new technologies to reduce fuel consumption and perform more efficient flights. In this scenario, time-related costs may play a role in the total flight costs, and thus the cost index adjustment may lead to faster flights. Additionally, newer aircraft may reach higher cruising speeds due to lower drag and more efficient engines. Expected sign: positive.

#### Market and industry environment

*GOL* and *AZUL* are dummy variables intended to empirically test the behavior of a low-cost airline regarding the determination of flight speed. Thus, it tests if these airlines prefer targeting higher aircraft utilization (faster flights) or if they prioritize fuel consumption reduction through slower and more economical flights. Note that Gol is notably viewed as the current Brazilian LCC. Azul has its origins associates with North American LCC Jetblue Airways. It initially had very low penetration prices in the industry but nowadays is considered for many the carrier with the highest yields and fares. Expected sign: undetermined.

*NCOMP* is the number of competing carriers on the route (in logarithm). If two carriers belong to the same parent company, we compute them as only one competitor. Expected sign: positive, as competition may for carriers to increase on-time performance to satisfy existing time-sensitive consumers. We also experiment with an alternative for *NCOMP*: the Herfindahl–Hirschman index (*HHI*), an index between 0 and 1. This variable indicates the level of market concentration on a given route in terms of RPK (Revenue Passenger Kilometer) calculated for each day (multiplied by 10,000, in logarithm). As with *NCOMP*, *HHI* index allows investigating the relationship between the competitiveness of a route and airlines' practices regarding flight speed. With these variables, we test the hypothesis that on routes with smaller *HHI*, i.e., with less market concentration, airlines perform higher speeds to ensure better on-time performance. On the other hand, on more concentrated routes, airlines could concern less about delays and focus on a more efficient operation in terms of fuel consumption^32^. Expected sign: negative.

*TREND* is a discrete variable set equal to {1, 2, 3, …} from the first operating day in the sample period. It allows capturing overall trend effects due to the dynamics of the industry business environment. Expected sign: undetermined.

*TREND* × *PAND* is equal to *TREND* multiplied by a dummy to account for the period of the COVID-19 pandemic (*PAND*), set equal to 1 since February 26, 2020, when the first coronavirus infection case was reported in Brazil. Expected sign: positive, as the period is marked by low demand and consequent airport and air traffic control idleness.

#### Unobserved factors

$u$ represents the error term of the regression models. As our data comprises a massive diversity of flights over thousands of days of operations, we believe that it is necessary to accommodate greater complexity related to the unobserved factors of decision-making regarding the flight speed of airlines. We thus assume a component error structure in $u$. In other words, we consider nuisance variables to accommodate idiosyncrasies related to flight operations, which, despite being observed by pilots and the airline, are not observable by the econometrician. We consider four versions of the proposed component error structure. First, we have the definition in Eq. (3):

3 $$u\equiv f\left({\xi }_{year},{\xi }_{month},{\xi }_{weekday}\right)+\upsilon ,$$

where ${\xi }_{year}$, ${\xi }_{month}$*,* and ${\xi }_{weekday}$ are fixed effects of year, month, and day of the week of the flight, respectively. In addition, $\upupsilon$ is a new random error term, which we assume to be normally distributed. For simplicity, we impose the additivity of function $f\left(.\right)$.

The other versions of the error term $u$ that we consider use route fixed effects. On account of that, we rename the error term to $\upomega$. The specification of $\upomega$ is expressed in Eq. (4):

4 $$\omega \equiv f\left({\xi }_{route},{\xi }_{year},{\xi }_{month},{\xi }_{weekday}\right)+\varepsilon ,$$

where ${\xi }_{route}$ are route fixed effects, and $\upvarepsilon$ is a random error term with the same properties as $\upsilon$. In this specification, we consider that there are idiosyncrasies related to the routes flown by airlines that exert influence on flight speeds variations observed in the data set, such as idiosyncrasies related to the geometry of the flight paths, the direction of the flight, and the characteristics of the terminal areas and endpoint airports.

One limitation of Eq. (4) is that the route effects do not allow considering season-specific variations in the data, such as the impact of typical seasonal wind direction, for example. To accommodate this option, a third version of $u$ is in Eq. (5):

5 $$\omega \equiv f\left({\xi }_{route-month},{\xi }_{year},{\xi }_{weekday}\right)+\varepsilon ,$$

where ${\xi }_{route-month}$ are route and month interacted fixed effects, that is, they accommodate route-to-route variations in each month of the year. See an alternative procedure for accommodating seasonal variation across routes in Eufrásio et al.^23^ and Oliveira et al.^32^. With this specification, each route now has time-specific variation across the year.

Finally, the fourth version of $u$ that we consider is expressed by Eq. (6):

6 $$\omega \equiv f\left({\xi }_{route-month},{\xi }_{year},{\xi }_{weekday},{\xi }_{aircraft model}\right)+\varepsilon ,$$

where ${\xi }_{aircraft model}$ are fixed effects of aircraft model type. With these fixed effects, it is possible to take into account the flight speed impacts of idiosyncrasies related to the engines and aerodynamics of each operated aircraft. For example, jet aircraft commonly fly at higher speeds than turboprop aircraft^36^.

The model specification contained in Eq. (2), combined with the simpler definition of $u$, expressed by Eq. (3), generates our baseline model. However, if we consider the versions of the component error dictated by Eqs. (4)–(6), we have that the perfect collinearity between the route-fixed effects and the distance variables makes the model estimation unfeasible. Therefore, when considering the more realistic models that include route fixed effects, or route-time effects, we do not utilize *VDIST* and *VDIST2*. However, route effects allow controlling for the factors associated with the distance between the pair of airports, in addition to other invariant factors in the temporal dimension. Thus, we reach Eq. (7), which contains our extended econometric model, considering any of the versions of $\upomega$:

7 $$SPD = {\beta }_{1}MASS+ {\beta }_{2}FUELP+ {\beta }_{3}DELDEP+ {\beta }_{4}DELARR+ {\beta }_{5}CONG+ {\beta }_{6}MAXFREQ+ {\beta }_{7}SLOT+ {\beta }_{8}HUB+ {\beta }_{9}AGE+ {\beta }_{10}NEWMOD+ {\beta }_{11}GOL+ {\beta }_{12}AZUL+ {\beta }_{13}NCOMP+ {\beta }_{14}TREND+ {\beta }_{15}TREND\times PAND+ \omega ,$$

where *SPD* = {*SPDMEA*, *SPDCRU*}. Our preferred specification combines Eqs. (6) and (7).

### Estimation strategy

To compute the estimation results, we employ the estimation procedure of linear regression absorbing multiple levels of fixed effects of Correia^37^, hereafter "MultLevFE". The standard errors of estimates are adjusted for route clusters.

In the ESM Appendix, we present a Table A1 with descriptive statistics of the model variables. We estimate a high correlation value between the variables *DELDEP* and *DELARR* of 0.9472—as expected, since delays in the departure may cause delays in the arrival time. Also expected is the relatively moderate correlation between *AGE* and *NEWMOD*, estimated as 0.3988. The pairwise correlations between the other covariates are typically below 0.25. Additionally, the estimated mean VIFs for the SPDMEA and SPDCRU models without fixed effects are low, equal to 2.72 and 3.06, respectively. Also, we identify the presence of heteroscedasticity through Breusch–Pagan/Cook–Weisberg tests. We, therefore, set the MultLevFE estimator to adjust the standard errors of estimates for route clusters.

As a set of robustness checks, we run a set of regressions considering the different possibilities dictated by Eqs. (2) and (7), and by the four versions of the proposed component error structure of Eqs. (3)–(6). We also experiment with *HHI* in substitution of *NCOMP* and discard the *TREND* and *TREND* × *PAND* variables in some specifications. We intend to observe how the results change by perturbations in the model. We report the results of the robustness checks along with our preferred regression results.

As a final set of robustness checks, we consider an alternative approach for building our regressands. We compute proxies for the deviation of observed flight speeds from the manufacturer recommended speeds. To compute such proxies, we calculate the "unimpeded speed", as defined by the US Federal Aviation Administration—see aspm.faa.gov/aspmhelp/index/ASPM_Arrival_Airport_Enroute__Definitions_of_Variables.html—which consists of the 90th percentile speed. We then compute the unimpeded flight speed versions for each aircraft model. Finally, we calculate the new variables *DSPDCRU* and *DSPDMEA*, consisting of the ratio between the corresponding flight speed and the unimpeded flight speed (in logarithm). We replace these alternative measures as the new regressands to assess the sensitivity of results and report them in the next section.

## Results and discussion

Tables 1 and 2 present the estimation results for the SPDCRU and SPDMEA models, respectively. In both tables, Column (6) shows the results of the preferred model, which includes all variables of Eq. (7), and all fixed effects dictated by Eq. (6). The estimation results of the remaining columns allow for an inspection of the robustness of our preferred model. The SPDMEA specification includes the flight delay management variables *DELDEP* and *DELARR*, while the SPDCRU specification does not. This is because SPDMEA represents the actual speed of the aircraft, while SPDCRU is a planned speed metric. Other differences are i. Table 1 does not incorporate aircraft model fixed effects, as its inclusion results in a high degree of multicollinearity in the SPDCRU models; and ii. to emulate the planning environment of the airline when setting SPDCRU, we compute the variables *MASS* and *CONG* in Table 1 as their respective sample means on the route over the previous 30 days, which results in *MASS [hist]* and *CONG [hist]*.

**Table 1:** Estimation results: flight planned cruise speed (*SPDCRU*, *DSPDCRU*).

Variables	(1) SPDCRU	(2) SPDCRU	(3) SPDCRU	(4) SPDCRU	(5) SPDCRU	(6) SPDCRU	(7) SPDCRU
Flight operations							
* VDIST*	1.3090***						
* VDIST2*	− 0.0983***						
* MASS [hist]*	0.4243***	0.0523***	0.0521***	0.0521***	0.0522***	0.0702***	− 0.0060
* FUELP*	0.0101	0.0062	0.0061	0.0061	0.0012	0.0007	0.0015
Airport operations							
* CONG [hist]*	− 0.0577***	− 0.0094***	− 0.0094***	− 0.0094***	− 0.0097***	− 0.0090***	0.0021
* MAXFREQ*	− 0.0085***	0.0025*	0.0025*	0.0025*	0.0024*	0.0024*	0.0007
* SLOT*	0.0201***	0.0027	0.0027	0.0027	0.0029	0.0040	− 0.0013
* HUB*	− 0.0299***	− 0.0042	− 0.0042	− 0.0042	− 0.0042	− 0.0045	− 0.0089
Aircraft factors							
* AGE*	0.0130***	0.0013	0.0013	0.0013	0.0013	0.0011	− 0.0015**
* NEWMOD*	0.0403***	0.0134***	0.0134***	0.0134***	0.0137***	0.0124***	0.0000
Market and industry environment							
* GOL*	− 0.0073	− 0.0133*	− 0.0132*	− 0.0132*	− 0.0133*	− 0.0128*	− 0.0345***
* AZUL*	− 0.0020	− 0.0477***	− 0.0477***	− 0.0477***	− 0.0478***	− 0.0467***	− 0.0393***
* HHI*	0.0316***	− 0.0091**					
* NCOMP*			0.0083**	0.0083**	0.0083**	0.0086**	− 0.0033*
* TREND*				− 0.0002	0.0016	0.0009	− 0.0082***
* TREND* × *PAND*					− 0.0004***	− 0.0004***	− 0.0002**
Estimator	MultLevFE	MultLevFE	MultLevFE	MultLevFE	MultLevFE	MultLevFE	MultLevFE
Fixed effects							
[1] Route	*No*	*Yes*	*Yes*	*Yes*	*Yes*	*No*	*No*
[2] Year	*Yes*	*Yes*	*Yes*	*Yes*	*Yes*	*Yes*	*Yes*
[3] Month	*Yes*	*Yes*	*Yes*	*Yes*	*Yes*	*No*	*No*
[4] Weekday	*Yes*	*Yes*	*Yes*	*Yes*	*Yes*	*Yes*	*Yes*
[5] Route-month	*No*	*No*	*No*	*No*	*No*	*Yes*	*Yes*
[6] Aircraft model	*No*	*No*	*No*	*No*	*No*	*No*	*No*
Clusters	Route	Route	Route	Route	Route	Route	Route
Adj. R^2^ statistic	0.6806	0.8855	0.8855	0.8855	0.8856	0.8945	0.5676
RMSE statistic	0.0900	0.0539	0.0539	0.0539	0.0539	0.0517	0.0314
No. observations	887,500	887,494	887,494	887,494	887,494	887,330	887,330

Results produced by linear regression absorbing multiple levels of fixed effects (MultLevFE) of Correia^37^. Std. errors adjusted for route clusters. The no. of observations across columns may vary due to singleton drops. p-value representations: ***p < 0.01, **p < 0.05, *p < 0.10.

Significant values are in [italics].

**Table 2:** Estimation results: flight mean speed (*SPDMEA*, *DSPDMEA*).

Variables	(1) SPDMEA	(2) SPDMEA	(3) SPDMEA	(4) SPDMEA	(5) SPDMEA	(6) SPDMEA	(7) SPDMEA
Flight operations							
* VDIST*	1.5770***						
* VDIST2*	− 0.0910***						
* MASS*	0.0040	0.0034*	0.0034	0.0033	0.0034	0.0035	0.0034*
* FUELP*	− 0.0007	− 0.0144***	− 0.0143***	− 0.0157***	− 0.0119***	− 0.0128***	− 0.0137***
Flight delay management							
* DELDEP*	0.7394***	0.7397***	0.7397***	0.7397***	0.7397***	0.7402***	0.7397***
* DELARR*	− 0.7325***	− 0.7338***	− 0.7338***	− 0.7338***	− 0.7338***	− 0.7342***	− 0.7337***
Airport operations							
* CONG*	− 0.0021***	− 0.0007***	− 0.0007***	− 0.0007***	− 0.0007***	− 0.0007***	− 0.0007***
* MAXFREQ*	− 0.0326***	− 0.0079***	− 0.0079***	− 0.0079***	− 0.0077***	− 0.0078***	− 0.0075***
* SLOT*	0.0090*	0.0060*	0.0061*	0.0060*	0.0061*	0.0062*	0.0072**
* HUB*	− 0.0358***	0.0018	0.0017	0.0017	0.0018	0.0018	0.0022
Aircraft factors							
* AGE*	− 0.0000	0.0001	0.0001	0.0001	0.0001	0.0001	0.0001
* NEWMOD*	0.0179***	0.0171***	0.0171***	0.0170***	0.0165***	0.0166***	0.0155***
Market and industry environment							
* GOL*	0.0388*	0.0030	0.0032	0.0033	0.0038	0.0040	− 0.0078
* AZUL*	− 0.0176***	− 0.0234***	− 0.0234***	− 0.0234***	− 0.0235***	− 0.0235***	− 0.0121
* HHI*	0.0156***	0.0019					
* NCOMP*			− 0.0022	− 0.0021	− 0.0021	− 0.0019	− 0.0011
* TREND*				0.0149***	0.0114***	0.0118***	0.0134***
* TREND* × *PAND*					0.0009***	0.0008***	0.0008***
Estimator	MultLevFE	MultLevFE	MultLevFE	MultLevFE	MultLevFE	MultLevFE	MultLevFE
Fixed effects							
[1] Route	*No*	*Yes*	*Yes*	*Yes*	*Yes*	*No*	*No*
[2] Year	*Yes*	*Yes*	*Yes*	*Yes*	*Yes*	*Yes*	*Yes*
[3] Month	*Yes*	*Yes*	*Yes*	*Yes*	*Yes*	*No*	*No*
[4] Weekday	*Yes*	*Yes*	*Yes*	*Yes*	*Yes*	*Yes*	*Yes*
[5] Route-month	*No*	*No*	*No*	*No*	*No*	*Yes*	*Yes*
[6] Aircraft model	*Yes*	*Yes*	*Yes*	*Yes*	*Yes*	*Yes*	*Yes*
Clusters	Route	Route	Route	Route	Route	Route	Route
Adj. R^2^ statistic	0.9002	0.9314	0.9314	0.9314	0.9314	0.9318	0.7143
RMSE statistic	0.0796	0.066	0.066	0.066	0.066	0.0658	0.0661
No. observations	6,060,062	6,060,052	6,060,052	6,060,052	6,060,052	6,059,834	6,059,834

Results produced by linear regression absorbing multiple levels of fixed effects (MultLevFE) of Correia^37^. Std. errors adjusted for route clusters. The no. of observations across columns may vary due to singleton drops. p-value representations: ***p < 0.01, **p < 0.05, *p < 0.10.

Significant values are in [italics].

In Column (1) we present a model without route-fixed effects, which allows us to estimate the coefficients of the flight distance variables *VDIST* and *VDIST2*. In Columns (2) to (5), in addition to year, month, and weekday fixed effects, we also include route fixed effects, and in Columns (6) and (7), route-month fixed effects. Due to perfect collinearity with the route (and route-month) fixed effects, *VDIST* and *VDIST2* cannot be estimated in these specifications. In Column (3), we replace the *HHI* variable with *NCOMP*. In Column (4) we include *TREND* and in Column (5) we have *TREND* × *PAND*. Finally, in Column (7) of both tables, we present specifications replacing the original regressands with the alternative measures of flight speed relative to unimpeded speed, respectively, DSPDCRU and DSPDMEA.

When we compare the results, we can pinpoint notable similarities and dissimilarities across Tables 1 and 2. The covariates that produce similar impacts on SPDCRU and SPDMEA across tables are *VDIST*, *VDIST2*, *CONG*, *NEWMOD*, and *AZUL*. In general, these estimated coefficients have the same sign over the specifications and are statistically significant at a 1% level. As expected, *VDIST* and *VDIST2* have positive and negative estimated coefficients, respectively, intuitively suggesting that flight speed increases at a decreasing rate with flight distance. *CONG* has negative estimated coefficients in most cases, indicating that airport congestion is an effective obstacle to airline flight operations optimization, both at planning and tactical management levels. *NEWMOD* has positive coefficients in most cases. This result provides evidence that newer-generation aircraft, known to be more economical, can also reach higher flight cruise and actual mean flight speeds. The results for *CONG* and *NEWMOD* have therefore important policy implications concerning aircraft fuel burn, costs, and passenger satisfaction.

Finally, *AZUL* has negative estimated coefficients. In principle, such a result only documents that this airline operates smaller aircraft with less powerful engines—E-Jets and turboprops—than the airline in the base case of the dummy, namely Latam Airlines. However, this result holds even in Table 2, where we also control for the fixed effects of the different aircraft models. An alternative explanation could therefore come from the fact that this carrier operates several domestic routes as a monopoly^38^, which would reduce the necessary market incentives to increase the speed of flights to satisfy its time-sensitive customers. However, in all specifications, we control for the effect of the market structure either with *HHI* or *NCOMP*. We, therefore, conclude that this result may be due to a specificity of the airlines' business model, which has its origins linked to North American LCC Jetblue Airways. The results have important managerial implications suggesting that the carrier's notable success in avoiding nonstop competition in domestic markets may confer the necessary peace of mind for planning and putting into practice flight speeds that are closer to the most fuel-efficient speed of its fleet's airplanes.

*HUB* also presents similar results across the estimation tables, but in this case, revealing non-statistically significant coefficients in most columns.

Another category of the similar result between Tables 1 and 2 concerns estimated coefficients that were not statistically significant in most cases or were significant only in isolated situations. The *SLOT*, *AGE*, *GOL*, *HHI*, and *NCOMP* regressors fit into this category. In these cases, we conclude that the impact of these variables on flight speed is either null or has limited evidence. For example, the variable *GOL* presents ambiguous results regarding a possible effect concerning the lower flight speed of this airline when confronted with the base case of the dummy (Latam Airlines). Although we find limited evidence suggesting that the carrier operates lower cruising speeds (SPDCRU), the results also suggest that this practice is not able to influence its actual mean speed (SPDMEA). We therefore cannot conclude that the major Brazilian LCC presents any different flight-speed setting behavior when compared to the major national FSC carrier.

Another example in this type of similarity across the tables is *NCOMP*, which seems to be positive and significant for SPDCRU, but barely significant for DSPDCRU—Column (7) of Table 1- and not significant at all for SPDMEA and DSPDMEA—both in Table 2.

Regarding the situations in which Tables 1 and 2 disagree, we identify the divergent results of the covariates *MASS*, *FUELP*, *MAXFREQ*, *TREND*, and *TREND* × *PAND*. The estimated coefficients of *FUELP* are almost always negative and statistically significant in the SPDMEA model, but not in SPDCRU. This finding suggests that pilots may use their aircraft's flight management system to adapt the actual flight speed to suit the cost conditions of the specific flight. On the other hand, it appears that the airline does not take into account such information when determining its planned cruise speeds for flights via the cost index settings.

The estimated coefficients of *MASS* are in most cases positive in SPDCRU, but not significant at all in SPDMEA. We, therefore, have evidence that the pressure to reduce flight times in situations of higher passenger and cargo demand is taken into account during the flight planning stage, but is apparently ignored by pilots. Another possibility is that in-flight speed adjustments are fully mitigated by the unobserved airport and air traffic conditions that may be correlated with *MASS*. In this case, there would be an endogeneity of the *MASS* variable, which would limit the interpretation of our results. We suggest further investigation into such a methodological issue.

The *MAXFREQ* coefficients are always negative and statistically significant in SPDMEA, but are generally not significant in SPDCRU. This result suggests that the scale of airport operations—as measured by *MAXFREQ*—can indeed impact the mean actual speeds, but this effect is not incorporated into the flight speed planning by airlines. We have important policy implications in these results, where we suggest that carriers add this factor to their flight planning to make the plans more consistent with actual operations.

Regarding the estimated trends, the results for *TREND* suggest a long-term gradual increase in mean speeds, but not in planned cruise speeds. Additionally, *TREND* × *PAND* suggests that such a trend was intensified for SPDMEA after the pandemic, implying that the lower airline demand in the period allowed airlines to operate increasingly faster flights. However, inconsistently, the *TREND* × *PAND* coefficient is negative for SPDCRU, pointing to a lack of coordination between the operations and planning tasks in the period. Again, we suggest that an effort be made to reconcile these two perspectives of company management, aiming at optimizing overall operational performance in the industry.

Finally, regarding *DELDEP* and *DELARR*, we confirm the initial assumptions raised. First, concerning *DELDEP*, the results indicate that the longer the departure delay, the greater the mean speeds, possibly in an attempt of the pilot to recover the schedule. For *DELARR*, there is an inverse relationship, i.e., higher arrival delays *TREND* to reduce mean speeds, probably due to air traffic control management and landing airport operations interference.

## Conclusion

This study empirically investigated the determinants of flight speeds of commercial aircraft operating in Brazil. We analyzed a vast dataset comprising millions of domestic flights over 14 years. Using an econometric approach, we developed a framework to study the planned cruise speed and the mean actual flight speed set by airlines.

Our findings corroborate that airport congestion is a formidable barrier to optimizing airline flight operations. Additionally, we confirm that newer-generation aircraft can attain higher cruise and mean actual flight speeds.

Concerning the impact of fuel cost conditions, our results suggest that pilots may use their aircraft's flight management system to adapt the actual flight speed to suit the economic environment of the date of the operations. In contrast, we find evidence that the airline does not consider such information when determining its planned cruise speeds for flights. We pinpoint other cases in which we suggest the airlines should make an effort to coordinate flight planning and operations more consistently.

Finally, regarding airlines' business models, our results do not allow us to conclude that the major Brazilian LCC Gol shows a different flight-speed setting pattern when compared to the major national full-service carrier. However, we find suggestive evidence that Azul airlines, which has its roots in US low-cost airline Jetblue Airways may employ lower planned and actual speeds, possibly targeting more fuel-efficient operations.

The findings of our empirical investigation into the determinants of flight speeds in Brazil hold significant policy implications. Specifically, we believe that our results have the potential to aid airlines in optimizing flight operations by facilitating a more streamlined and efficient management of aircraft fuel consumption, costs, and passenger satisfaction.

## Supplementary Information

Supplementary Information.

## Data Availability

To guarantee our results’ reproducibility, we uploaded the survey dataset at https://doi.org/10.7910/DVN/UCAZ1S.
